# Supplementary material for: Heterogeneity in M. tuberculosis β-lactamase inhibition by Sulbactam
Source: Nat Commun. 2023 Sep 7;14:5507. doi: 10.1038/s41467-023-41246-1 (PMC10485065; doi:10.1038/s41467-023-41246-1)
Supplement: Supplementary file 1 — Supplementary Information [file 41467_2023_41246_MOESM1_ESM.pdf]

Supplementary Material for

Heterogeneity in *M. tuberculosis* β-Lactamase Inhibition by Sulbactam

Tek Narsingh Malla[1], Kara Zielinski[2], Luis Aldama[3], Sasa Bajt[4,5], Denisse Feliz[3], Brendon Hayes[6], Mark Hunter[6], Christopher Kupitz[6], Stella Lisova[6], Juraj Knoska[5], Jose Martin-Garcia[7],Valerio Mariani[6], Suraj Pandey[1], Ishwor Poudyal[1], Raymond G. Sierra[6], Alexandra Tolstikova[8], Oleksandr Yefanov[5], Chung Hong Yoon[6], Abbas Ourmazd[1], Petra Fromme[9], Peter Schwander[1], Anton Barty[8,10], Henry N. Chapman[4,5,11], Emina A. Stojkovic[3], Alexander Batyuk[6], Sébastien Boutet[6], George N. Phillips, Jr.[12,13], Lois Pollack[2], Marius Schmidt[1*]

[1] Physics Department, University of Wisconsin-Milwaukee, 3135 N Maryland Ave, Milwaukee, WI 53211, USA

[2] School of Applied and Engineering Physics, Cornell University, 254 Clark Hall, Ithaca, NY 14853, USA

[3] Department of Biology, Northeastern Illinois University, 5500 N. St. Louis Ave., Chicago, Illinois 60625, USA

[4]The Hamburg Centre for Ultrafast Imaging, Luruper Chaussee 149, 22761 Hamburg, Germany

[5]Center for Free-Electron Laser Science CFEL, Deutsches Elektronen Synchrotron, Notkestrasse 85, 18 22607 Hamburg, Germany

[6]Linac Coherent Light Source LCLS, SLAC National Accelerator Laboratory, 2575 Sand Hill Road, Menlo Park, CA 94025, USA

[7] Department of Crystallography and Structural Biology, Institute of Physical Chemistry, Rocasolano, Spanish National Research Council (CSIC), Serrano 119, 28006 Madrid, Spain

[8] Deutsches Elektronen-Synchrotron DESY, Notkestrasse 85, 22607 Hamburg, Germany

[9] School of Molecular Sciences and Biodesign Center for Applied Structural Discovery, 20 Arizona State University, Tempe, AZ 85287-1604, USA

[10]Center for Data and Computing in Natural Science CDCS, Deutsches Elektronen-Synchrotron DESY, Notkestrasse 85, 22607 Hamburg, Germany

[11]Department of Physics, Universität Hamburg, Luruper Chaussee 149, 22761 Hamburg, Germany

[12]Department of BioSciences, Rice University, 6100 Main Street, Houston, Texas 77005, USA

[13]Department of Chemistry, Rice University, 6100 Main Street, Houston, Texas 77005, USA

* corresponding author: smarius@uwm.edu

This PDF file includes:

Supplementary Figures 1 - 3

Supplementary Tables 1 - 4

Supplementary Notes

Supplementary References

Supplementary Figures

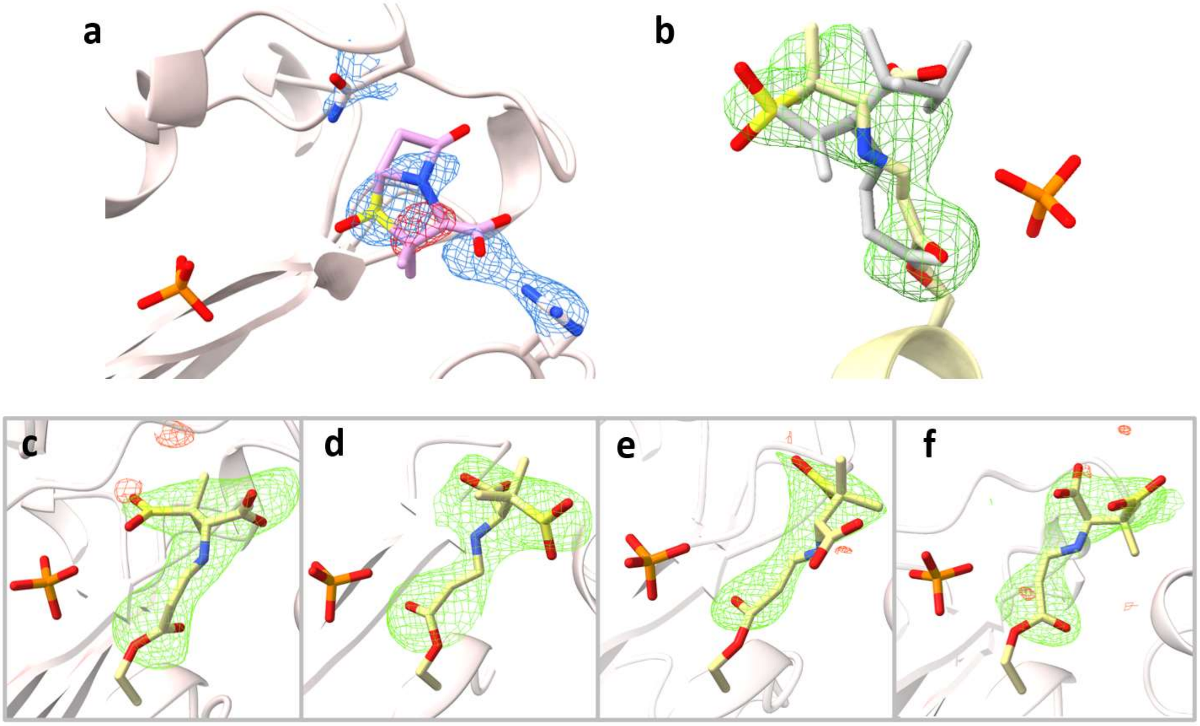

Supplementary Figure 1. (a) The appearance of SUB at the entrance of the active site in subunit A. SUB (shown in purple) was placed in the DED_{omit} feature near the guardian residues observed at Δt_{misc} = 3 ms and refined. After refinement negative density (red contour lines) appear in the F_{obs}-F_{calc} map (displayed on the 3σ level). The B-factors of all SUB atoms are in excess of 120 $Å^2$. Translational and rotational disorder of the free SUB near the guardian residues renders the refinement of a single conformation difficult. (b) *Cis*-enamine (shown in grey) poorly fits the electron density at Δt_{misc} = 15 ms (shown as an omit map. (c-f) Polder omit maps, contoured at 3σ, determined in each subunit of BlaC (A - D), respectively, after 3 h of soaking with SUB. All the species are characterized by covalently bound TENs.

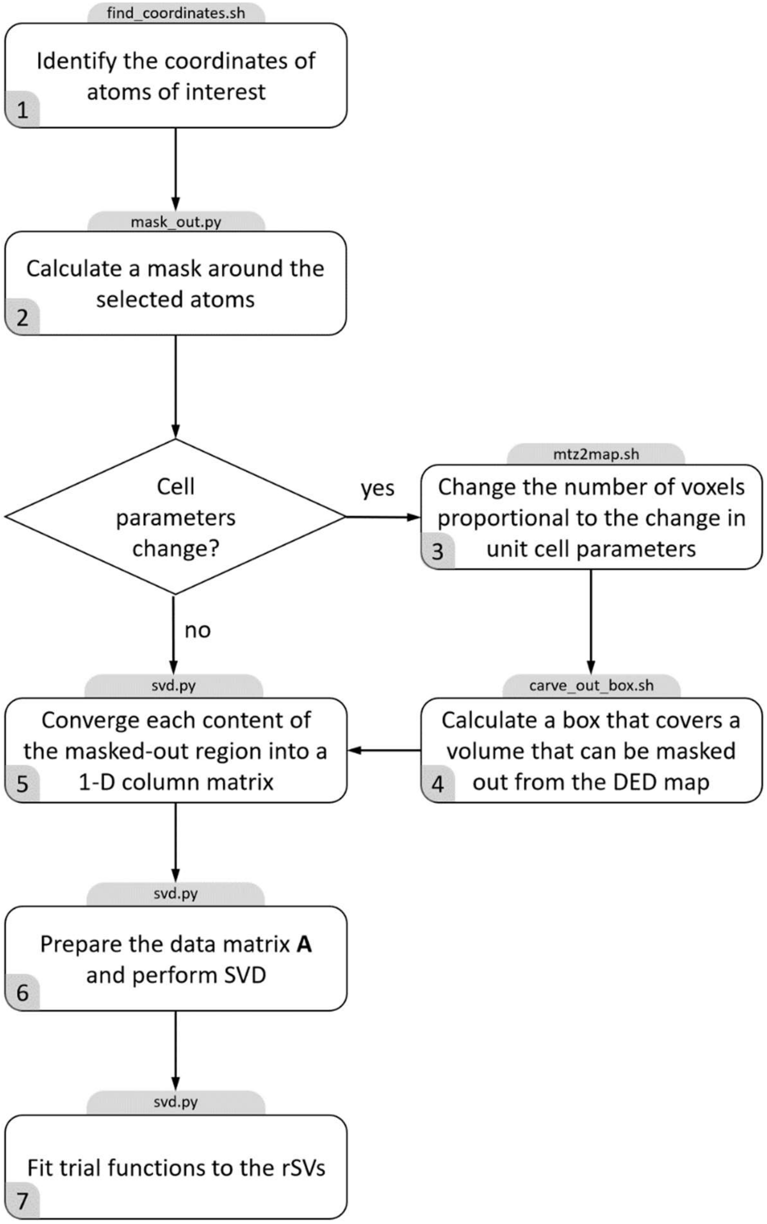

Supplementary Figure 2: Flow chart for an SVD analysis of time-dependent crystallographic difference maps. The grey boxes on top are the scripts/programs developed and used to accomplish each step.

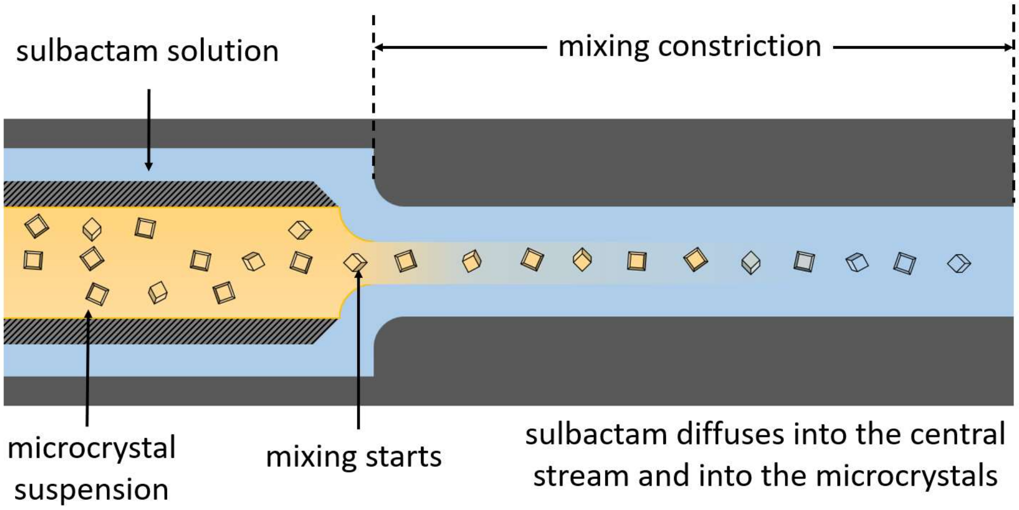

Supplementary Figure 3. Cartoon cross section of the injector focused on the mixing region adapted from Calvey et al.[74]. Randomly oriented BlaC microcrystals flow through an inner capillary, whereas the sulbactam solution flows concentrically through the outer capillary. Both flows are combined into a single outlet of reduced diameter called the mixing constriction. The time taken by crystals to move from the start of the mixing constriction until they are injected into the X-ray interaction region determines the MISC time delay. The sulbactam diffuses into the central flow containing crystals. ~100 mM of ligand concentration in the central flow is reached after 15 ms.

Supplementary Tables

Supplementary Table 1: Data collection and refinement statistics

	Water	3ms	6ms	15ms	30ms	240ms	700ms	3 h soak
**Data collection**								
Space group						$P2_1$		
a,b,c (Å), β (°)	79.6,98.1, 110.9,108.5	80.5,98.8, 111.5,108.5	80.4,98.8, 112.1,108.7	80.3,98.6, 112.1,108.8	80.2,98.5, 112.3,108.9	79.8,97.9, 113.2,109.3	80.0,98.3, 112.1,108.8	79.4, 96.7, 111.1,108.5
Resolution range (Å)	22.14-2.2 (2.3 -2.2)†	22.01-2.6 (2.75-2.6)	20.53-2.75 (2.85-2.75)	20.86-2.65 (2.75-2.65)	20.4-2.95 (3.06-2.95)	22.42-2.35 (2.43-2.34)	20.94-3.2 (3.38-3.2)	22.3-2.7 (2.8-2.7)
Hits‡	88,978	61,508	26,309	51,166	27,143	75,038	2,854	n.a
Indexed patterns	73,965	36,400	17,596	31,958	11,123	59,928	2,740	n.a
Observed reflections	37,857,581	14,718,802	6,258,604	11,092,596	3,888,789	29,990,504	1,582,566	n.a
Unique reflections	83,447	42,822	43,284	40,586	32,517	68,441	27,514	38,773
Multiplicity	453(335)	343(164)	144(69)	273(130)	119(67)	446(306)	57(35)	2.7 (1.3)
Completeness	100(100)	100(100)	100(100)	100(100)	100(100)	100(100)	99.9(99.9)	79(28)
$R_{split/merge}$ (%)	23.3(528)	23.4(427)	24.3(354)	23.6(316)	30.4(176)	28.8(689)	44.6(208)	18.2(803)
CC* (%)	99.6(69.8)	99.3(55.9)	99.0(51.6)	98.9(58.7)	98.2(52.9)	99.3(64.2)	95.9(51.3)	99.6(64.2)
**Refinement**								
Resolution range	22.14-2.2	22.01-2.6	20.53-2.75	20.86-2.65	20.4-2.95	22.42-2.35	20.94-3.2	22.3-2.7
Reflections used	68064	40968	41929	38742	33082	53680	27127	25,400
R_{cryst}/R_{free}	0.22/0.25	0.22/0.24	0.22/0.24	0.23/0.26	0.22/0.26	0.23/0.25	0.27/0.29	0.22/0.27
Occupancy (in %)	n.a	n.a.	n.a.	TEN[B]: 96<br>TEN[D]: 90	SUB[A]: 52<br>TEN[B]: 85<br>SUB[C]: 63<br>TEN[D]: 77	TEN[A]: 100<br>TEN[B]: 100<br>TEN[C]: 100<br>TEN[D]: 100	TEN[A]:100<br>TEN[B]:100<br>TEN[C]:100<br>TEN[D]:100	TEN[A]:98<br>TEN[B]:96<br>TEN[C]:98<br>TEN[D]:88
r.m.s.d‡ bond length (Å)	0.004	0.002	0.003	0.003	0.003	0.003	0.002	0.003
r.m.s.d bond angles (°)	0.641	0.545	0.601	0.642	0.768	0.65	0.428	0.584
No. of H_2O	447	140	203	183	44	311	16	90

† Values in parentheses represent the highest resolution bin

‡ In serial crystallography, a hit is defined as the image potentially containing Bragg reflections

A,B,C,D The superscript letters represent corresponding subunits.

Supplementary Table 2: Important distances (in Å) in the active centers of subunits A and B

	water	3ms	6ms	15ms	30ms	240ms	700ms
(a) Distance between side chain of some active residues in subunit A							
$Ser70^A$ OG to $Arg173^A$ CZ	10.9	11.6 (2.7)†	12.1 (3.1)	10.7 (1.2)	11.0 (1.6)	10.4 (1.4)	10.2 (1.3)
$Ser70^A$ OG to $Gln112^B$ CD	10.6	12.0 (2.2)	10.9 (0.4)	10.9 (1.3)	10.6 (0.4)	9.9 (0.5)	9.8 (0.2)
$Ser70^A$ OG to $Gln109^B$ CD	9.0	10.2	10.0	10.3	10.2	8.6	8.7
(b) Distance between side chains of some active residues in subunit B							
$Ser70^B$ OG to $Arg173^B$ CZ	13.9	13.2 (2.1)	13.9 (1.7)	14.4 (1.6)	14.8 (0.9)	14.9 (1.5)	14.4 (1.2)
(c) Distances from BlaC side chains to selected SUB and TEN atoms in subunit A							
$Thr239^A$ O to SUB^A O12	-	-	-	-	2.7	-	-
$Gln112^B$ NE2 to SUB^A O8	-	-	-	-	2.7	-	-
$Lys73^A$ NZ to TEN^A O8	-	-	-	-	-	2.5	3.1
$Glu168^A$ OE2 to TEN^A O8	-	-	-	-	-	2.6	2.5
$Asn172^A$ ND2 to TEN^A O12	-	-	-	-	-	2.5	2.9
(d) Distances from BlaC side chains to selected TEN atoms in subunit B							
$Thr239^A$ O to TEN^B O8	-	-	-	2.9	3	3.1	3.2
$Gln109^A$ NE2 to TEN^B O12	-	-	-	3.9	3.4	3.2	4.1

†Values in parentheses represent the change in the position [in Å] of the terminal side chain atom from its original position at reference structure.

A,B subunits that host the amino acid.

Supplementary Table 3: Relaxation times and amplitudes determined from fitting Eqn. 2 to the significant rSVs

	**Subunit A**	**Subunit B**	**Subunit C**	**Subunit D**
λ (ms^{-1})	0.25	0.51	0.33	0.69
τ_1 (ms)	23.26	10.17	25.53	9.81
τ_2 (ms)	86.03	80.41	90.01	88.15
**rSV_1**				
$A_{0,1}$	11.72	38.32	8.89	32.77
$A_{1,1}$	118.95	195.44	113.3	155.37
$A_{2,1}$	134.48	122.15	152.08	137.01
**rSV_2**				
$A_{0,2}$	-17.96	-0.25	-12.95	3.23
$A_{1,2}$	-90.67	-41.79	-74.92	26.54
$A_{2,2}$	142.88	64.27	117.31	-48.72

λ, τ_1 and τ_2 are global fit parameters for all significant rSVs; the amplitudes ‘A’s are determined independently for each rSV.

Supplementary Table 4: Injector geometry and sample flow rates

	Water	Sulbactam					
Δt_{misc} (ms)	0	3	6	15	30	240	700
Ligand concentration (mM)	n.a.	150					
Ligand buffer	water	50 mM Ammonium Phosphate (pH 4.5)					
Injector ID	3	1	1	2	2	3	4
Crystal flow rate (µl/min)	10	7.7	7.6	7.7	11.1	10.1	11
Ligand flow rate (µl/min)	70	142.3	67.4	143.1	63.1	70	57
Constriction inner diameter (µm)	100	50	50	50	50	100	100
Constriction length (mm)	82.7	10.3	10.3	40.7	40.7	82.7	35‡
Timing uncertainty (ms)	n.a.	±1.0	±2.0	±1.1	±3.5	±18.8	±29.5
Ligand concentration at the end of constriction (mM)	n.a.	58.5	58.5	100.5	88.5	112.5	99

‡For timepoints longer than 500 ms, an additional delay stage is added immediately after the constriction.

Supplementary Notes

Sulbactam forms a *trans*-enamine complex with BlaC

The acyl moieties are known to tautomerize into more stable *cis*- or *trans*-enamine products. In accordance, the SUB bifurcates into *cis*- and *trans*-enamines caused by the isomerization about the C5=C6 double bond [1,2] (Fig. 1 d, e). The presence of both products has been reported from spectroscopic data favoring the *trans*-enamine form[3,4]. It is argued that the further isomerization of the *cis*- to the *trans*- form is promoted by intrinsic steric clashes of the *cis*- form [5,6]. At the resolution achieved here (between 2.35 Å and 3.2 Å, Supplementary Table 1), it is important to correctly discriminate between these two forms. The carboxylic and sulfinic moieties branch out from the common stem but they would form a similar shaped electron density in either of the conformations. To address this concern, a *cis*-enamine was placed in the DED_{omit} maps and refined (Supplementary Fig. 1 b). The model poorly fitted the DED, and worse R-factors were obtained. This shows that the *trans*-enamine is the only species that can explain the electron density properly.

Supplementary References

1. Zhu, F., Li, R., Feng, D. C., He, M. X. & Cai, Z. T. Theoretical studies of sulbactam: Reactions after acylation. *Int J Quantum Chem* **107**, 1925-1934, doi:10.1002/qua.21307 (2007).
2. Li, R., Liao, J. M., Gu, C. R., Wang, Y. T. & Chen, C. L. Theoretical investigation on reaction of sulbactam with wild-type SHV-1 beta-lactamase: acylation, tautomerization, and deacylation. *The journal of physical chemistry. B* **115**, 10298-10310, doi:10.1021/jp111572v (2011).
3. Totir, M. A., Helfand, M. S., Carey, M. P., Sheri, A., Buynak, J. D., Bonomo, R. A. & Carey, P. R. Sulbactam forms only minimal amounts of irreversible acrylate-enzyme with SHV-1 beta-lactamase. *Biochemistry* **46**, 8980-8987, doi:10.1021/bi7006146 (2007).
4. Kalp, M., Totir, M. A., Buynak, J. D. & Carey, P. R. Different intermediate populations formed by tazobactam, sulbactam, and clavulanate reacting with SHV-1 beta-lactamases: Raman crystallographic evidence. *Journal of the American Chemical Society* **131**, 2338-2347, doi:10.1021/ja808311s (2009).
5. Padayatti, P. S., Helfand, M. S., Totir, M. A., Carey, M. P., Carey, P. R., Bonomo, R. A. & van den Akker, F. High resolution crystal structures of the trans-enamine intermediates formed by sulbactam and clavulanic acid and E166A SHV-1 {beta}-lactamase. *The Journal of biological chemistry* **280**, 34900-34907, doi:10.1074/jbc.M505333200 (2005).
6. Kalp, M., Bethel, C. R., Bonomo, R. A. & Carey, P. R. Why the extended-spectrum beta-lactamases SHV-2 and SHV-5 are "hypersusceptible" to mechanism-based inhibitors. *Biochemistry* **48**, 9912-9920, doi:10.1021/bi9012098 (2009).
