# Supplementary material for: Heterogeneity in M. tuberculosis β-lactamase inhibition by Sulbactam
Source: Nat Commun. 2023 Sep 7;14:5507. doi: 10.1038/s41467-023-41246-1 (PMC10485065; doi:10.1038/s41467-023-41246-1)
Supplement: Supplementary file 3 — Description of Additional Supplementary Files [file 41467_2023_41246_MOESM3_ESM.docx]

**Description of additional Supplementary items**

**Supplementary Movie 1: Sulbactam binding and reaction with subunit A of the BlaC**

Zooming into the occluded active site of subunit A (purple) after comparison with the open active site of subunit B (red). The MISC time-delays are shown on the lower right. The non-covalent binding of an intact SUB is shown that further reacts to a covalently bound TEN species. The movie has been assembled by interpolation (morphing) between the measured structures at 3 ms, 6 ms, 15 ms, 30 ms, 66 ms, 240 ms and 700 ms. Trajectories between the measured species are, therefore, artificial and should be regarded with caution as a guide to the eye.

**Supplementary Movie 2: The opening of the active site in subunit A**

Van-der-Waals surface representation of the entrance to the subunit A active site (purple) that is occluded by residues of subunit B (red). The movie has been assembled by interpolation (morphing) between the measured structures at 3 ms, 6 ms, 15 ms, 30 ms, 66 ms, 240 ms and 700 ms. The perceived dynamics is, therefore, artificial and should be regarded with caution as a guide to the eye.
