# Supplementary material for: Heterogeneity in M. tuberculosis β-lactamase inhibition by Sulbactam
Source: Nat Commun. 2023 Sep 7;14:5507. doi: 10.1038/s41467-023-41246-1 (PMC10485065; doi:10.1038/s41467-023-41246-1)
Supplement: Supplementary file 6 — Reporting Summary [file 41467_2023_41246_MOESM6_ESM.pdf]

nature portfolio

Corresponding author(s): Schmidt, Marius

Last updated by author(s): Aug 9, 2023

Reporting Summary

Nature Portfolio wishes to improve the reproducibility of the work that we publish. This form provides structure for consistency and transparency in reporting. For further information on Nature Portfolio policies, see our Editorial Policies and the Editorial Policy Checklist.

Statistics

For all statistical analyses, confirm that the following items are present in the figure legend, table legend, main text, or Methods section.

n/a	Confirmed	
☒	☐	The exact sample size (*n*) for each experimental group/condition, given as a discrete number and unit of measurement
☒	☐	A statement on whether measurements were taken from distinct samples or whether the same sample was measured repeatedly
☒	☐	The statistical test(s) used AND whether they are one- or two-sided<br>*Only common tests should be described solely by name; describe more complex techniques in the Methods section.*
☒	☐	A description of all covariates tested
☒	☐	A description of any assumptions or corrections, such as tests of normality and adjustment for multiple comparisons
☒	☐	A full description of the statistical parameters including central tendency (e.g. means) or other basic estimates (e.g. regression coefficient) AND variation (e.g. standard deviation) or associated estimates of uncertainty (e.g. confidence intervals)
☒	☐	For null hypothesis testing, the test statistic (e.g. *F*, *t*, *r*) with confidence intervals, effect sizes, degrees of freedom and *P* value noted<br>*Give P values as exact values whenever suitable.*
☒	☐	For Bayesian analysis, information on the choice of priors and Markov chain Monte Carlo settings
☒	☐	For hierarchical and complex designs, identification of the appropriate level for tests and full reporting of outcomes
☒	☐	Estimates of effect sizes (e.g. Cohen's *d*, Pearson's *r*), indicating how they were calculated

Our web collection on statistics for biologists contains articles on many of the points above.

Software and code

Policy information about availability of computer code

Data collection	Room temperature data was collected at the MFX instrument of the LCLS using the ePixl0k detector. OnDa (Online Data Analysis) Monitor (OM) was used for live monitoring of the data collection process. Cheetah was used as the preliminary hit-finder for the XFEL data. Both are open source software and the manuals are available online.<br>Cryo temperature data was collected at beamline 19-ID-D at APS. Data was collected with proprietory sbccollect program installed at APS.
Data analysis	Data were analyzed by CrystFEL version 0.9.1, CCP4 version 7.1, coot version 0.9.5, phenix version 1.19.2-4158, and by self developed python (version 3.8) and bash scripts. The self written programs are explained in detail in the methods section of the manuscript. Python code and shell scripts are available on GitHub [https://github.com/73km/pySVD4TX] and on zenodo [https://doi.org/10.5281/zenodo.8206588].

For manuscripts utilizing custom algorithms or software that are central to the research but not yet described in published literature, software must be made available to editors and reviewers. We strongly encourage code deposition in a community repository (e.g. GitHub). See the Nature Portfolio guidelines for submitting code & software for further information.

Data

Policy information about availability of data

All manuscripts must include a data availability statement. This statement should provide the following information, where applicable:

- Accession codes, unique identifiers, or web links for publicly available datasets
- A description of any restrictions on data availability
- For clinical datasets or third party data, please ensure that the statement adheres to our policy

All relevant data are included in the paper or Supplementary information and/or available from corresponding author upon reasonable request. The structure factors and the refined coordinates of the XFEL structure of BlaC mixed with sulbactam for 3 ms, 6 ms, 15 ms, 30 ms, 240 ms, 700 ms and 3 hours (the cryo-soaked structure) have been deposited in the Protein Data Bank (PDB) database under accession codes 8GCV, 8GCS, 8GCT, 8EBI, 8EBR, 8EC4, 8GCX and 8ECF respectively. Atomic coordinates of BlaC in other crystal forms used for comparison in this study are available in the PDB under accension codes 5OYO and 7A71. Source data are provided with this paper.

Research involving human participants, their data, or biological material

Policy information about studies with human participants or human data. See also policy information about sex, gender (identity/presentation), and sexual orientation and race, ethnicity and racism.

Reporting on sex and gender	n/a
Reporting on race, ethnicity, or other socially relevant groupings	n/a
Population characteristics	n/a
Recruitment	n/a
Ethics oversight	n/a

Note that full information on the approval of the study protocol must also be provided in the manuscript.

Field-specific reporting

Please select the one below that is the best fit for your research. If you are not sure, read the appropriate sections before making your selection.

☒ Life sciences ☐ Behavioural & social sciences ☐ Ecological, evolutionary & environmental sciences

For a reference copy of the document with all sections, see nature.com/documents/nr-reporting-summary-flat.pdf

Life sciences study design

All studies must disclose on these points even when the disclosure is negative.

Sample size	Sample size is not pre calculated for serial crystallography experiments. Instead data is collected until the signal is noticeably stronger than the noise for each dataset. As such, the sample size for each dataset is different. All the resulting statistical data on the number of crystals and the quality of data is reported in Supplementary Table 1.
Data exclusions	A large amount of images are recorded in a serial crystallography experiment. The images without Bragg reflections do not contribute to crystallographic analysis and thus excluded from analysis.
Replication	Millions of diffraction patterns were collected over a course of 4 days at LCLS. Out of them, approximately 325,000 diffraction patterns had real Bragg reflection and were used to determine the results. The robustness and reliability of the result were tested using various established quality factors, such as the R-split, the CC-half, etc.
Randomization	The orientation of microcrystals in serial crystallography is random by nature. Each diffraction pattern is collected from a randomly oriented crystal. Hundreds of thousands of these diffraction pattern are collected for every data set. These randomized patterns are processed to obtain end results. The quality is calculated from randomly subsampled data. As is standard in crystallography, the 5% of the reflection were chosen randomly to calculate Rfree to assess the quality of the structure generated upon refinement.
Blinding	Blinding is not applicable in this experiment as the conditions of data collection depend on the pre-determined time point. Each time is a separate data set. Even so, investigators have no control which crystal in which orientation result in the diffraction pattern.

Reporting for specific materials, systems and methods

We require information from authors about some types of materials, experimental systems and methods used in many studies. Here, indicate whether each material, system or method listed is relevant to your study. If you are not sure if a list item applies to your research, read the appropriate section before selecting a response.

Materials & experimental systems

n/a	Involved in the study
☒	☐ Antibodies
☒	☐ Eukaryotic cell lines
☒	☐ Palaeontology and archaeology
☒	☐ Animals and other organisms
☒	☐ Clinical data
☒	☐ Dual use research of concern
☒	☐ Plants

Methods

n/a	Involved in the study
☒	☐ ChIP-seq
☒	☐ Flow cytometry
☒	☐ MRI-based neuroimaging
